# Education and training in radiation protection in Europe: results from the EURAMED Rocc-n-Roll project survey

**DOI:** 10.1186/s13244-023-01398-6

**Published:** 2023-04-01

**Authors:** Joana Santos, Shane Foley, Jonas Andersson, João Paulo Figueiredo, Christoph Hoeschen, John Damilakis, Guy Frija, Francisco Alves, Katrine Riklund, Louise Rainford, Ursula Nestle, Jonathan McNulty, Klaus Bacher, Monika Hierath, Graciano Paulo

**Affiliations:** 1 Instituto Politécnico de Coimbra, ESTESC - Coimbra Health School, Medical Imaging and Radiotherapy, Rua 5 de Outubro, S. Martinho Do Bispo, 3046-854 Coimbra, Portugal; 2 Radiography and Diagnostic Imaging, School of Medicine, University College Dublin, Dublin, Ireland; 3 Department of Radiation Sciences, Radiation Physics, Umeå University, Umeå, Sweden; 4 Institute of Medical Technology, Otto-von-Guericke University Magdeburg, Magdeburg, Germany; 5 School of Medicine, University of Crete, Iráklion, Crete, Greece; 6 Université de Paris, Paris, France; 7 Department of Radiation Sciences, Diagnostic Radiology and Umeå Centre for Functional Brain Imaging, Faculty of Medicine, Umeå University, Umeå, Sweden; 8 Department of Radiation Oncology, Kliniken Maria Hilf, Mönchengladbach, Germany; 9 Department of Radiation Oncology, University Hospital Freiburg, Freiburg, Germany; 10 Division of Medical Physics, Department of Human Structure and Repair, Ghent University, Ghent, Belgium; 11 European Institute for Biomedical Imaging Research (EIBIR), Vienna, Austria

**Keywords:** Radiation protection, Education and training, European Basic Safety Standards, Medical exposure

## Abstract

**Purpose:**

To analyse the existing radiation protection (RP) education and training (E&T) capabilities in the European Union and identify associated needs, problems and challenges.

**Method:**

An online survey was disseminated via the EURAMED Rocc-n-Roll consortium network and prominent medical societies in the field of radiological research. The survey sections analyse the RP E&T during undergraduate, residency/internship and continuous professional development; RP E&T problems and legal implementation. Differences were analysed by European geographic regions, profession, years of professional experience and main area of practice/research.

**Results:**

The majority of the 550 respondents indicated that RP topics are part of undergraduate curricula in all courses for their profession and country (55%); however, hands-on practical training is not included according to 30% of the respondents. The lack of E&T, practical aspects in current E&T, and mandatory continuing E&T were considered the major problems. The legal requirement that obtained higher implementation score was the inclusion of the practical aspects of medical radiological procedures on education (86%), and lower score was obtained for the inclusion of RP E&T on medical and dental school curriculums (61%).

**Conclusions:**

A heterogeneity in RP E&T during undergraduate, residency/internship and continuous professional development is evident across Europe. Differences were noted per area of practice/research, profession, and European geographic region. A large variation in RP E&T problem rating was also obtained.

## Key points

1. European health professionals reveal different RP E&T experiences.
2. Around 12% of the respondents report the absence of undergraduate RP topics.
3. The radiation protection problems perception varies across Europe.
4. The results revealed a heterogeneity of compliance with the EU recommendations.

## Introduction

The development of medicine and technology has considerably increased the use of ionising radiation for diagnostic proposes in recent decades [1]. Despite this evolution, large variations in European radiation protection (RP) education and training (E&T) for health professionals have been reported [2–6]. In order to harmonise RP E&T, the European Commission in 2012 established guidelines, which were published in the European Commission report RP No. 175 [7]. More recently, in 2018, with the transposition of the European Basic Safety Standards (BSS) Directive 59/2013, member states are required to promote and define RP E&T for health professionals employed in various fields [8].

The EURAMED Rocc-n-Roll project was a three-year initiative to achieve a European strategic research agenda (SRA) in the field of medical applications with ionising radiation and related RP aspects. In the project, a comprehensive analysis was performed on medical RP research, innovation, and related E&T identified to generate the largest benefit for the European population. The project also focused on including safety and quality aspects throughout Europe, while fostering clinical translation, strengthening economic growth and industrial competitiveness. The objective of EURAMED Rocc-n-Roll Work Package 7 (WP7) was to develop a methodological framework and guidance document on how to organise, implement and disseminate medical RP E&T amongst health professionals. Furthermore, the WP7 framework also included E&T for research projects to ensure researchers have a sound knowledge base of the science required for RP research, to build capacity in RP research and ensure sustainability of the field, including strengthening links with industry regarding new technological developments, from a RP perspective. The methodological framework is intended to serve as a foundation for a strategy to establish a harmonised and sustainable safety culture in RP amongst health professionals and researchers, as well as engaging new generations in this field of research [9]. A specific aim of WP7 (7.1) was to analyse the existing RP E&T capabilities in the European Union (EU) and identify the needs, problems, and challenges for each health profession in relation to the use of ionising radiation in medicine and related RP E&T. To fulfil this aim, characterisation of the implementation status, at national level, of the requirements regarding E&T defined in the European BSS Directive was also performed [7, 8].

## Methods

An online survey was created in Google forms (Google LLC, Menlo Park, USA), based on the European Commission report RP Report No. 175 [7], the Ibero-American Conference on RP in Medicine (CIPRAM) report [10] and the European BSS Directive [8], and iteratively improved by all WP7 members.

The survey was evaluated as a pilot by the Rocc-n-Roll advisory board and external experts (*n* = 14) in November 2020, and the main survey was initiated in February 2021. The survey was sent to professional and scientific societies in Europe from the fields of medical imaging, nuclear medicine, and radiation oncology (*n* = 13), as well as to European platforms such as the European Radiation Dosimetry Group (EURADOS), Multidisciplinary European Low Dose Initiative (MELODI) and European Alliance Medical Radiation Protection Research (EURAMED), and related European projects. The survey was open for nine weeks, with reminders sent to all potential respondents after four weeks.

The survey was divided into six sections covering social and demographic data, RP E&T undergraduate curricula, RP E&T during residency/internship (where applicable), RP E&T as part of continuing professional development (CPD), frequency of RP E&T possible problems based on a list of statements, and characterisation of the level of implementation of BSS in daily practice. A summary of the survey is presented in Appendix A. Ethical approval was obtained and written informed consent was achieved from all subjects that responded to the survey.

To better organise respondents’ replies, *professions* were re-organised to facilitate statistical analyses (from 21 to 13 options); respondents were either contacted directly or their online profiles were analysed and classified based on their ORCID records (where available). The option *other physician* was created to integrate other physicians, i.e. *non-radiologists*, *nuclear medicine* or *radiation oncologist*.

Statistical analysis was performed using IBM® SPSS® Statistics software version 26.0 (IBM SPSS Inc., Chicago, USA). Differences between respondents’ replies were analysed by European regions, profession, years of professional experience, and main area of practice or research. To analyse significant differences based on European regions, an additional variable was created based on EuroVOc [11], which is a multilingual lexicon maintained by the Publications Office of the EU. The country variable was maintained and a variable with four region options was defined as Northern Europe, Western Europe, Southern Europe, and Central and Eastern Europe. Following a descriptive analysis of years of professional experience, respondents were grouped using the SPSS visual binning tool. SPSS software proposed five groups: [< = 5], [6–16], [17–27], [28 to 38], and [> = 39], which were used in the analysis of respondents’ replies. The main five areas of practice or research were selected for further analysis and statistical tests. Descriptive statistical analysis of frequency and percentage of RP E&T problem rating was performed. The perception of the level of implementation of BSS on the daily practice was analysed based on five statements, and results were converted to scores from 0 to 100. Pearson’s Chi-Square tests, Fisher’s exact tests, Mann–Whitney *U* test, T-Student tests, Kruskal Wallis test (with Post Hoc test Bonferroni) and one way ANOVA tests were used to analyse significant differences between respondents as appropriate. A 95% confidence interval was used for inferential statistics.

## Results

A total of 550 survey responses were obtained, 58% of respondents were male (*n* = 317). Most respondents were in the age range 46–55 years (33%), followed by 36–45 years (30%), and over 56 years (25%).

### Characterisation of social demographic data

**Fig. 1:**
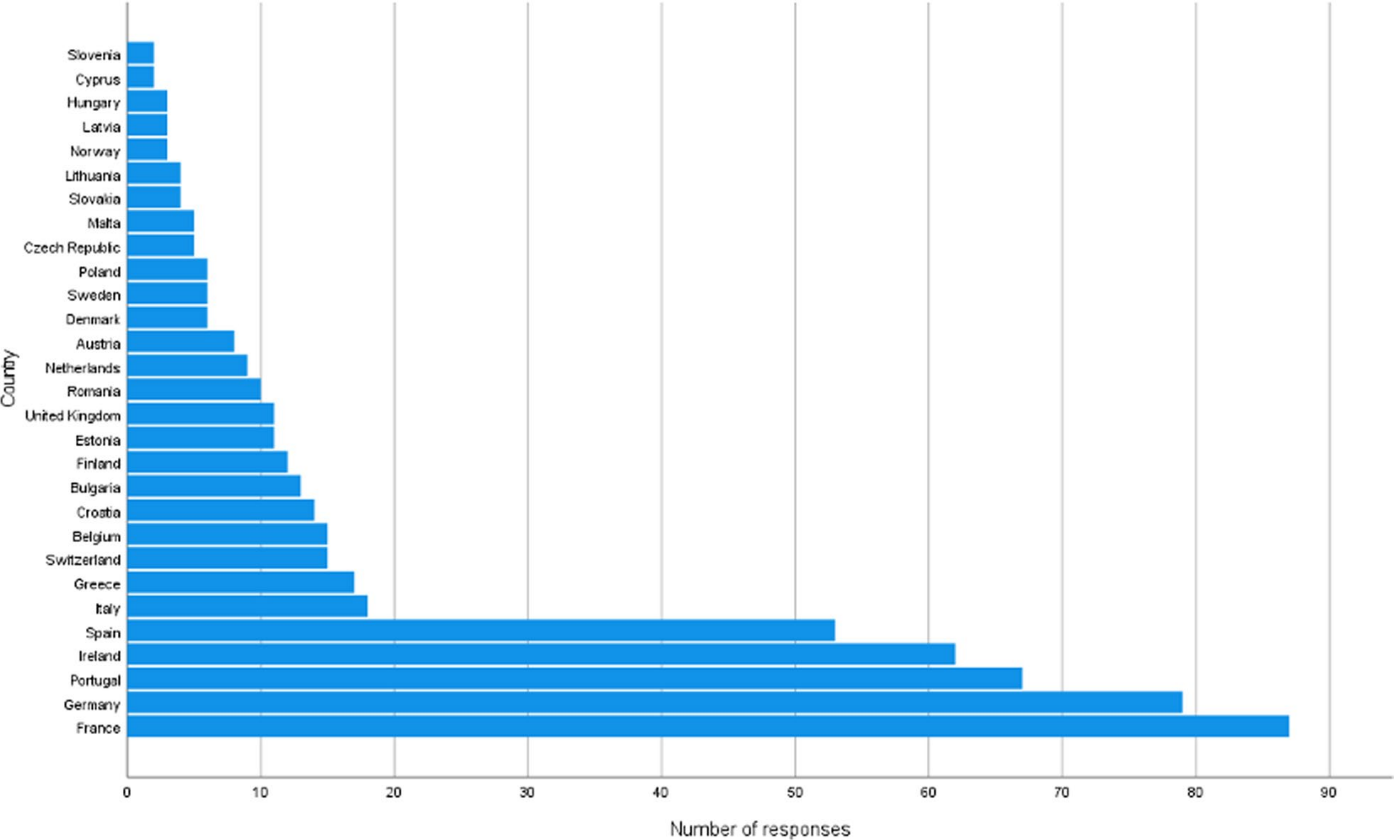
Number of replies per country

The distribution of respondents per country is shown Fig. 1. The highest frequency of responses was obtained from France (16%), Germany (14%) and Portugal (12%).

The region of Western Europe returned the highest number of responses (49%, *n* = 271), followed by Southern Europe (30%, *n* = 162), and Central and Eastern Europe (13%, *n* = 72). The Northern Europe region presented the lowest number of responses (8%, *n* = 45).

Table 1 describes the frequency and percentage of the respondents’ profession. Amongst respondents, medical physicists (28%) and radiographers (22%) were the most common professions.

**Table 1:** Profession frequency and percentage of the respondents

Profession	*n*	%
Dentist	46	8.4
Medical physicist	151	27.5
Nuclear medicine physician	45	8.2
Other physician	10	1.8
Radiation biologist	19	3.5
Radiation oncologist	50	9.1
RP expert	21	3.8
Radiographer	118	21.5
Radiologist	66	12.0
Radiopharmacist	3	< 1
Regulator	12	2.2
Research in RP & medical physics	9	1.6
Total	550	100.0

The mean years of professional experience of respondents was 21 (standard deviation 10) years. The highest frequency of responses came from those with a 17–27 years of experience range (38%, *n* = 206), followed by 6–16 years (31%, *n* = 166), and 28–38 years (21%, *n* = 115). Most respondents indicated, through multiple choice, that they work in a hospital or teaching hospital (60%, *n* = 330), followed by educational institution and hospital (11%, *n* = 65).

The top five were *Diagnostic Radiology* (41%, *n* = 227), *Radiotherapy* (17%, *n* = 93), *Medical Imaging and Radiotherapy* (13%, *n* = 69), *Nuclear Medicine* (11%, *n* = 60), and *Dental Imaging* (6%, *n* = 31).

### RP in E&T undergraduate curricula

Table 2 presents the results of the inclusion of RP topics as part of undergraduate curricula and inclusion of hands-on RP E&T topics.

**Table 2:** Characterisation of the presence of RP topics in undergraduate curricula and presence of hands-on E&T RP topics

Undergraduate curricula E&T RP topics	Present		Hands-on	
	*n*	%	*n*	%
Yes, in all	304	55.3	119	22.6
Yes, in some	155	28.2	212	40.2
No	65	11.8	156	29.6
Don’t Know	26	4.7	40	7.6
Total	550	100.0	527	100.0

Most respondents indicated that RP topics were a part of undergraduate curricula in all the courses for their profession and country (55%). Significant differences were found per reported profession (*p* = 0.013). *Nuclear Medicine physicians*, *Radiation Oncologists*, *Radiologists*, and *Regulators* presented similar values for “*Yes, in all*” and “*Yes, in some*” and Medical Physicists (18%) and Nuclear Medicine physicians (18%) presented higher values for “*No*”. Significant differences were also found per area of practice or research (*p* = 0.012). The *Radiotherapy* and *Medical Imaging and Radiotherapy* areas of practice or research presented higher values for “*Yes, in some*” (34% and 33%, respectively) and “*No*” (16% and 18%, respectively) (Table 3).

**Table 3:** Characterisation of the presence of RP topics on residency/internship and presence of hands-on E&T RP topics

Residency/intership E&T RP topics		Present		Hands-on	
		*n*	%	*n*	%
Yes	In all	243	53.5	295	66.3
	In some	154	33.9		
No		38	8.4	109	24.5
Don’t Know		19	4.2	41	9.2
Total		454	100.0	445	100.0

According to survey respondents, most professions consider that RP topics were included in all the undergraduate courses. However, *Nuclear Medicine Physicians* (36%), *Radiation Oncologists* (40%) and *Regulators* (42%) consider that this occurs in some of the undergraduate courses. The professionals that practice or research in the fields of *Medical Imaging and Radiotherapy* (50% “*Yes, in all*”, 34% “*Yes, in some*” and 16% *“No”*) and *Radiotherapy* (50% “*Yes, in all*”, 33% “*Yes, in some*” and 18% *“No”*) presented higher variation of opinion in comparison with the other professional areas. No significant differences were found per EU regions or years of professional experience.

Only 23% of respondents reported to have hands-on practical training included in all RP undergraduate curricula, with 40% answering “Yes, in some” and 30% stated that such training was not included. Significant differences were found per area of practice or research (*p* < 0.0001). While most *Dental Imaging* professionals reported having hands-on training during undergraduate curricula (64%), *Radiotherapy* (55%), *Medical Imaging and Radiotherapy* (52%) and *Nuclear Medicine* professionals presented higher values (46%) for the “*Yes, in some*” option. The area of practice or research with the largest variation of responses was *Diagnostic Radiology* with 26% for “*Yes, in some*”, 38% for “*Yes, in all*” and 36% of “*No*”. No significant differences were found according to the categories of EU region, profession, or professional experience in years. Nevertheless, for these categories, a large proportion of the respondents answered “*Yes, in some*” (40%).

The majority (62%) of respondents rated E&T in RP in undergraduate curricula as “*very good*” or *“adequate”.* However, 28% of respondents considered it “*insufficient”,* and some reported that no RP topics were included (6%). Statistically significant differences were identified per area of practice or research (*p* < 0.0001). All reported professional areas, excepting *Radiotherapy* presented high levels of satisfaction on this subject, with more than 60% of responses categorised as “very good” or “adequate”. Almost half of the *Radiotherapy* respondents (46%) rated E&T in RP as either insufficient or none.

### RP in E&T during the residency/internship

A sizeable number of respondents (17%) did not respond to questions related to residency/internship, which may be related to the EU professionals that do not need to follow a residency/internship programme to achieve a professional license. More than half of the 454 respondents answered that RP topics are included in all the residency/internship programmes (54%).

Respondents’ replies had no significant differences regarding the presence of RP topics during residency/internship per *EU region*, *Professionals*, *Professional experience in years*, or *Area of practice or research*. Considering the EU region, a similar number of responses was obtained for “*Yes, in all*” and “*Yes, in some*” for Central Eastern Europe (47% and 45%, respectively) and Northern Europe (46% and 49%). Similar values for “*Yes, in all*” were obtained for *Radiation oncologists* (49%), *Radiologists* (47%) and *Regulators* (50%). The “*Yes, in some*” responses reveal a variation across the residency/internship courses to achieve a professional license. While most respondents (66%) reported having hands-on practical training during their residency/internship to access a professional license, a quarter (25%) of respondents indicated having no such training included.

Significant differences were found per area of practice or research (*p* = 0.011) on the inclusion of hands-on practical training indicated by the survey respondents. Most respondents (66%) had hands-on practical training included during their residency/internship. However, respondents from *Diagnostic Radiology* (29%) and *Nuclear Medicine* (33%) indicated that no such RP training was included in their residency/internship.

No significant differences were obtained per EU region, Profession, or Number of years of professional experience. However, when analysed within each profession, some respondents (around 30% in some professions “Other Physician”, “Radiation Biologist”, “Radiation Oncologist” and “Medical Physicist”) indicated that hands-on RP E&T is not a requirement during residency/internship to access a professional license.

Respondents’ satisfaction with E&T in RP during residency/internship was evaluated, where it was found that a majority (54%) considered it “*adequate*”, while 20% categorised such E&T as “*very good*”. Significant differences were found per area of practice or research (*p* = 0.028). A large proportion of *Nuclear Medicine* (89%), *Radiotherapy* (79%) and *Medical Imaging and Radiotherapy* (80%) professionals (per area of practice/research) rated E&T during residency/internship as adequate and very good. For Diagnostic Radiology, more than 30% of respondents considered the RP E&T during residency/internship as “*insufficient*” or not “*included*”.

When data were analysed per EU Region, profession, and years of professional experience, most respondents (54%) rated RP E&T during residency/internship as “*adequate*”. For Radiographers, the most frequent responses were “adequate” (48%) and “insufficient” (32%).

### RP in E&T during continuing professional development

The need for mandatory RP E&T courses after entering a profession, as part of CPD was evaluated, with most respondents (68%) indicating that RP E&T CPD courses are mandatory. Significant differences were found per Profession (*p* = 0.019), where most respondents indicated that such CPD courses are mandatory CPD (values higher than 60%). However, a considerable number of *Other physicians* (60%), *Medical physicists* (37%), *Radiographers* (35%) and *Nuclear Medicine physicians* (33%) reported not having mandatory CPD courses within RP E&T. Significant differences were also found within respondents’ area of practice or research, where those from *Dental Imaging* that indicated to not have RP E&T included as mandatory CPD courses (*p* < 0.0001).

The periodicity of CPD was analysed based on the options “annually”, “every 2–3 years”, “every 4–5 years” and “every 6 or more years”. Significant differences in the number of years between CPD were obtained for the categories of Professionals (*p* = 0.046) and Area of practice/research (*p* < 0.0001). Radiation Biologists mostly indicated that they have CPD every 2 to 3 years (47%), in contrast to respondents from other professions who mostly indicated their CPD as every 4 to 5 years: *RP Expert* (67%), *Regulato*r (60%), *Radiation Oncologist* (59%), *Medical Physicist* (52%), *Radiopharmacist* (50%), *Other Physician* (50%), *Dentist* (47%), *Nuclear Medicine Physician* (45%), *Radiologist* (44%) and *Radiographer* (38%). Per *Area of practice or research*, most respondents from *Diagnostic Radiology* (49%) and *Nuclear Medicine* (48%) answered that they have mandatory CPD courses “annually” or “every 2 to 3 years”. The respondents from *Radiotherapy* (70%), *Dental Imaging* (55%) and *Medical Imaging and Radiotherapy* (53%) mostly answered that they have mandatory CPD courses every 4 to 5 years. No significant differences in respondents’ replies were found per EU region or *Years of professional experience*.

The respondents’ attendance at CPD courses in RP was analysed, and most indicated that they attended a CPD course in RP within the last three years (78%). Significant differences on the attendance frequency in the last three years were found Per area of practice or research (*p* = 0.001). The professional area with the highest frequency of RP CPD attendance was *Medical Imaging and Radiotherapy* (89%) and the lowest was *Dental Imaging* (54%).

The analysis of respondents hands-on practical training in CPD programmes including RP indicated that only 34% of the respondent’s performed a CPD programme with hands-on practical training included. Significant differences were found for EU region (*p* = 0.007), where most of the Northern Europe respondents had hands-on training included (58%), contrary to the results from other EU regions. No significant differences were found for the other sociodemographic topics.

### Rating of RP E&T problems

A list of 17 statements (S1-S17) were presented to respondents to rate, with respondents’ reflections on these statements shown in terms of frequencies and percentages in Table 4.

**Table 4:**
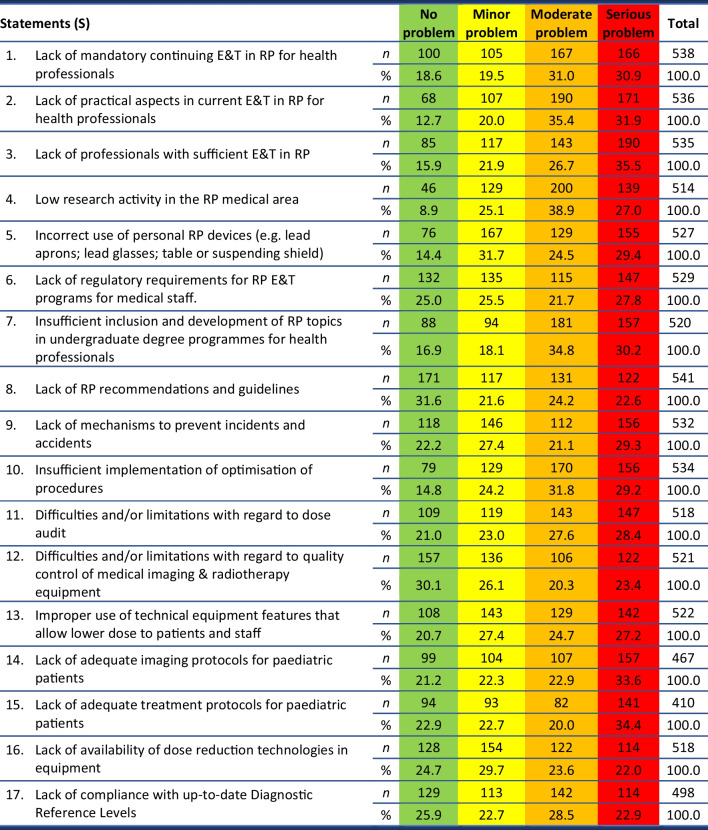
RP problems rated as “no problem” (green), “minor problem” (yellow), “moderate problem” (orange) and “serious problem” (red)

Significant differences per EU region were found for the scores of the following statements:

- S8 Lack of RP recommendations and guidelines (*p* = 0.020);
- S15 Lack of adequate treatment protocols for paediatric patients (*p* = 0.033).

These statements were considered more problematic for respondents from *Northern Europe*, when compared with responses from the other EU regions respondents. The majority of *Southern Europe* (*p* = 0.032) respondents considered the “*Lack of RP recommendations and guidelines*” as “no problem”. Around 50% of the respondents from *Western Europe* (*p* = 0.037) considered the “*Lack of adequate treatment protocols for paediatric patients*” as “no problem” or “minor problem”.

Significant differences per profession were found for *other physicians* (*n* = 10) and the *Radiopharmacist* (*n* = 3) on statements S1, S3, S9, S10 and S11.

Significant differences per *Area of practice or research* were found for the following statements:

- S1 Lack of mandatory continuing E&T in RP for health professionals (*p* = 0.026)—differences were found for *Dental Imaging* and *Diagnostic Radiology* (*p* = 0.009); and for *Radiotherapy* and *Diagnostic Radiology* (*p* = 0.013);
- S2 Lack of practical aspects in current E&T in RP for health professionals (*p* = 0.022)—differences were found for *Dental Imaging* and *Diagnostic Radiology* (*p* = 0.007); *Dental Imaging* and *Medical Imaging and Radiotherapy* (*p* = 0,004); and for *Radiotherapy* with *Medical Imaging* (*p* = 0.043);
- S4 Low research activity in the RP medical area (*p* = 0.013)—differences were found for *Dental Imaging* and *Diagnostic Radiology* (*p* = 0.034); *Nuclear Medicine* and *Diagnostic Radiology* (*p* = 0.006); and for *Radiotherapy* with *Diagnostic Radiology* (*p* = 0.019);
- S6 Lack of regulatory requirements for RP E&T programs for medical staff (*p* = 0.045)—differences were found for *Radiotherapy* and *Diagnostic Radiology* (*p* = 0.006); and for *Medical Imaging and Radiotherapy* and *Diagnostic Radiology* (*p* = 0.043);
- S7 Insufficient inclusion and development of RP topics in undergraduate degree programmes for health professionals (*p* = 0.003)—differences were found for *Dental Imaging* and *Radiotherapy* (*p* = 0.028); *Dental Imaging* with *Medical Imaging and Radiotherapy* (*p* = 0.011); *Dental Imaging* and *Diagnostic Radiology* (*p* < 0.0001); and for *Nuclear Medicine* and *Diagnostic Radiology* (*p* = 0.018);
- S11 Difficulties and/or limitations with regard to dose audit (*p* < 0.0001)—differences were found for *Radiotherapy* and *Medical Imaging and Radiotherapy* (p = 0.004); *Radiotherapy* and *Diagnostic Radiology* (*p* < 0.0001); *Dental Imaging* and *Medical Imaging and Radiotherapy* (*p* = 0.048); *Dental Imaging* and *Diagnostic Radiology* (*p* = 0.016); and for *Nuclear Medicine* and *Diagnostic Radiology* (*p* = 0.011).

### Level of implementation of the European BSS Directive in daily practice

Five statements on the implementation of RP legislation were presented to respondents, as shown in Table 5 together with the survey results.

**Table 5:** Characterisation of statements about BSS implementation

Statements about BSS (SBSS)		Don’t Know	Not implemented	Partially implemented	Fully Implemented
1 - Member States shall ensure that practitioners and the individuals involved in the practical aspects of medical radiological procedures have adequate education, information and theoretical and practical training for the purpose of medical radiological practices, as well as relevant competence in RP	*n*	36	48	259	207
	%	6.5	8.7	47.1	37.6
2 - Member States shall ensure that appropriate curricula are established and shall recognise the corresponding diplomas, certificates, or formal qualifications	*n*	58	61	214	217
	%	10.5	11.1	38.9	39.5
3 - Member States shall ensure that continuing E&T after qualification is provided	*n*	38	95	224	193
	%	6.9	17.3	40.7	35.1
4 - In the special case of the clinical use of new techniques. training is provided on these techniques and the relevant RP requirements	*n*	64	77	249	160
	%	11.6	14.0	45.3	29.1
5 - Member States shall encourage the introduction of a course on RP in the basic curriculum of medical and dental schools	n	124	90	203	133
	%	22.5	16.4	36.9	24.2

When the “partially” and “fully implemented” responses were analysed together, the highest frequency was obtained for SBSS 1 (85%), and the lowest frequency was obtained for SBSS 5 (61%). No significant differences were found across EU regions.

Significant differences were found for SBSS 1 (*p* = 0.022), SBSS 2 (*p* = 0.009), and SBSS 3 (*p* = 0.036). *Medical Physicists* and *Radiographers* presented higher frequency of responses on the options of “partially implemented” and “fully Implemented” (for SBSS 1 53% and 44%; and SBSS 2 56% and 45%; and SBSS 3 50% and 44%, respectively). For SBSS 3, *Radiologists* also presented higher values for the option “fully Implemented” (27%).

Significant differences were found for SBSS 1 (*p* = 0.022). Respondents with less than 5 years of professional experience presented a higher percentage of responses on the option “don’t know” (21%). However, respondents with more years of experience more frequently selected the options “partially” and “fully implemented”. For the other questions no significant differences were found per years of professional experience.

## Discussion

Despite RP E&T being recognised as crucial to optimise both patient and staff radiation doses while maintaining diagnostic and treatment requirements by the International Atomic Energy Agency (IAEA) [12], International Commission on Radiological Protection (ICRP) [13], World Health Organisation (WHO) [10], and the European Commission (EC) [7, 8], the results of the present work show a large variations in adoption amongst health professionals across the undergraduate courses. Notably 12% of the respondents to this survey reported the absence of RP topics as part of undergraduate curricula for their profession and country. This result reveals a lack of accordance with the recommendations of European Commission report RP No. 175 and the BSS Directive [7, 8]. A large variation in responses regarding undergraduate RP E&T was reported by professionals that practice or research in *Medical imaging and radiotherapy*. These results are in accordance with the European Federation of Radiographer Societies (EFRS) and European Federation of Organisations for Medical Physics (EFOMP) reports and recent studies published, that reveal a lack of harmonisation on undergraduate curricula across the Europe, despite the European BSS Directive and existence of guidelines for different professions working with ionising radiation applications in healthcare [4, 5, 14, 15].

In the report RP No. 175, the European Commission recommend a balance between education and training, where hands-on and problem solving is considered a pedagogical methodology to achieve student learning and course objectives [7]. Hands-on training is also mentioned, in the RP No. 175 “Accreditation, certification and recognition of medical education and training in RP” section, as an effective method to provide real-world experience, which ensures that a trainee carries out measurements and understands RP instead of just being instructed about RP theory and associated topics [7]. In the present survey, 84% of respondents reported having RP E&T topics included in the undergraduate curricula, 30% of these respondents reported the absence of hands-on in this field during undergraduate courses. This percentage is similar to that previously reported by the IAEA (35%) [16].

In the present study the top RP E&T identified problems were: the lack of E&T, lack of practical aspects in current E&T, and lack of mandatory continuing E&T. These identified problems are in agreement with the conclusions of a recent IAEA meeting [16], which identified similar weaknesses: heterogeneity on education level, insufficient or inadequate training in some medical professions, and lack of mandatory continuing E&T. The identified RP E&T possible problems were similar to the publication of the CIPRAM results. However, the CIPRAM report presented different problems per profession, despite the absence of problem rating [10].

The lack of adequate paediatric protocols for imaging and treatment was included in the survey results ranking of serious problems. According to the European Commission report RP 162 [17], the existence of paediatric protocols is mandatory for equipment acceptability and the BSS enforces this recommendation [8]. The EuroSafe Imaging and Image Gently campaigns reveal the concern for the use of adult protocols for paediatric examinations, as well as the usage of protocols with the incorrect paediatric categorisation, established in the European Commission report RP 185 [18], the problems identified in this study are similar to these concerns.

When the classifications of “moderate problem” and “serious problem” were merged, another problem appears as number 2 (66%) “low research activity in the RP medical area”. However, recently the European Strategic Research Agenda for RP developed by research platforms was discussed, a definition of joint research priorities was established and EURAMED Rocc-n-Roll will define a SRA and roadmap for medical applications of ionising radiation [19].

The results of the survey revealed that, in relation to implementation of the European BSS Directive, the statement reported as having the worst degree of implementation was “Member States shall ensure that continuing E&T after qualification is provided” (17%). This is in accordance with the 30% of the respondents who reported that CPD is not mandatory in their country for their chosen profession. These results are consistent with previously reported IAEA survey results [16].

The significant difference detected for the Northern Europe region was the inclusion of hands-on training during CPD courses without significant differences per respondents’ profession. In Western Europe, when the results were analysed based on profession, the inclusion of RP E&T topics during undergraduate curricula was statistically different (*p* = 0.018) for Medical Physics (15%), Nuclear Medicine Physician (22%) and Radiographers (18%). However, no differences were noted in Western Europe per profession, for RP E&T during residency/internship, hands-on during residency/internship, mandatory CPD and the inclusion of hands-on during CPD.

The Northern European respondents presented different perceptions about the lack of RP recommendations and guidelines compared to respondents from other regions (*p* = 0.020) and about the adequate treatment protocols for paediatric patients (*p* = 0.033). However, no significant differences were found per profession for these statements.

When the perception of the BSS implementation was analysed with the “partly implemented” and “fully implemented” responses merged, the highest percentage of implementation was obtained for “Member States shall ensure that practitioners and the individuals involved in the practical aspects of medical radiological procedures have adequate education, information and theoretical and practical training for the purpose of medical radiological practices, as well as relevant competence in RP” (85%); and the lowest percentage of implementation was obtained for “Member States shall encourage the introduction of a course on RP in the basic curriculum of medical and dental schools” (61%). These results are according to IAEA results and recent articles that identify lack of harmonisation on RP E&T on physicians and dentists [2, 6, 16].

The low frequency of responses and a higher number of responses from 5 countries can be considered as a limitation of the present European survey, that affected the data analyses per EU regions and for some professional groups, such as *Radiopharmacist*, *Research in RP & medical physics* and *other Physicians*.

It was difficult to clearly identify the needs and challenges of each health profession on RP E&T considering the heterogeneity of responses. However, the response distribution per profession was proportional to the profession rate per EU region.

Moreover, despite the low frequency of responses for some professions, the results obtained for those with higher number of responses gave the following feedback regarding the needs and challenges:

- *Nuclear Medicine physicians* (18%) indicated that RP topics were not a part of undergraduate curricula;
- *Radiation Oncologists* and *Medical Physicists* (≈30%) indicated that hands-on RP E&T is not a requirement during residency/internship to access a professional license;
- *Medical physicists* (37%), *Radiographers* (35%) and *Nuclear Medicine physicians* (33%) reported that there is no mandatory CPD courses within RP E&T.

## Conclusion

The survey results revealed different RP E&T experiences and problem perceptions across Europe. It is important to underline that 12% of the respondents to this survey report the absence of RP topics as part of undergraduate curricula for their profession and country. Around 28% of the respondents that had undergraduate RP E&T classified the topic as insufficient and 6% as not included. The results revealed a heterogeneity of compliance with RP 175 and the BSS Directive. Different perceptions of the possible RP E&T problems and lack of legislation implementation were identified per area of practice/research and EU regions.

## Data Availability

It is not possible to share research data publicly.

## Abbreviations

BSS: European Basic Safety Standards
CIPRAM: Ibero-American Conference on RP in Medicine
CPD: Continuing professional development
E&T: Education and training
EC: European Commission
EFOMP: European Federation of Organisations for Medical Physics
EFRS: European Federation of Radiographer Societies
EU: European Union
EURADOS: European Radiation Dosimetry Group
EURAMED: European Alliance Medical Radiation Protection Research
IAEA: International Atomic Energy Agency
ICRP: International Commission on Radiological Protection
MELODI: Multidisciplinary European Low Dose Initiative
RP: Radiation protection
SRA: European strategic research agenda
WHO: World Health Organisation
WP7: EURAMED Rocc-n-Roll Work Package 7

## References

[1] United Nations (2021) Sources, Effects and Risks of Ionizing Radiation UNSCEAR 2020/2021. Vol. Supplement, United Nations Scientific Committee on the Effects of Atomic Radiation. Available from: https://www.unscear.org/unscear/en/publications/2020_2021_3.html

[2] Ottolenghi A, Trott K, Baiocco G, Smyth V (2019). Education and training in Europe to support low-dose radiation physics and radiobiology. Radiat Prot Dosim.

[3] ESR E (2019) Patient Safety in Medical Imaging: a joint paper of the European Society of Radiology (ESR) and the European Federation of Radiographer Societies (EFRS). Insights Imaging. 10(1) 10.1186/s13244-019-0721-y PMC6449408 30949870

[4] EFRS. European Federation of Radiographer Societies (2018) European Qualifications Framework (EQF) level 6, Benchmarking Document: Radiographers, 2nd edn

[5] Maas AJJ, Lammertsma AA, Agius S, Bert C, Byrne B, Caruana CJ (2021). Education, training and registration of medical physics experts across Europe. Phys Med.

[6] Ploussi A, Efstathopoulos EP, Brountzos E (2021). The importance of radiation protection education and training for medical professionals of all specialties. Cardiovasc Intervent Radiol.

[7] European Commission (2014) Radiation Protection n^o^ 175, Guidelines On Radiation Protection Education And Training Of Medical Professionals In The European Union. Available from: https://ec.europa.eu/energy/sites/ener/files/documents/175.pdf

[8] European Commission. European Council Directive 2013/59/Euratom, on basic safety standards for protection against the dangers arising from exposure to ionising radiation and repealing [Internet]. 2014 p. ISSN 1977–0774. Available from: http://eur-lex.europa.eu/legal-content/EN/ALL/?uri=OJ:L:2014:013:TOC

[9] EURAMED 29 partners from 17 countries. EURAMED rocc-n-roll [Internet]. project funding from the Euratom research and training programme 2019–2020 under grant agreement No 899995. Available from: https://roccnroll.euramed.eu/about-rocc-n-roll/

[10] Consejo de Seguridad Nuclear (2017) Manuscripts published in a special issue of the journal RADIOPROTECCIÓN regarding the Ibero-American Conference on Radiation Protection in Medicine (CIPRaM). Available from: https://cdn.who.int/media/docs/default-source/documents/radiation/ibero-american-conference-on-radiation-protection-in-medicine-cipram.pdf?sfvrsn=1054bf38_5

[11] Union E (2021) EUR-Lex Acess to European Union law. Available from: https://eur-lex.europa.eu/browse/eurovoc.html?params=72,7206#arrow_7206

[12] IAEA (International Atomic Energy Agency) (2019) Postgraduate Educational Course in Radiation Protection and the Safety of Radiation Sources - Standard Syllabus. Train Course Ser No 18 (Rev 1). 18. Available from: https://www-pub.iaea.org/MTCD/Publications/PDF/TCS-18-Rev.1_web.pdf

[13] ICRP. Recommendations of the International Commission on Radiological Protection - Education and Training in Radiological Protection for Diagnostic and Interventional Procedures. Vol. Ann. ICRP, ICRP Publication 113. 2009. Available from: https://www.icrp.org/publication.asp?id=ICRP Publication 113 10.1016/j.icrp.2011.01.002 21788173

[14] Weiss S, Van Herzeele I (2020). Radiation protection training for vascular surgeons in twenty-one European Countries. Eur J Vasc Endovasc Surg.

[15] Foley S, Paulo G, Vassileva J (2022). Large differences in education and training of radiographers in Europe and Central Asia: results from an IAEA coordinated study. Radiography.

[16] Vassileva J, Applegate K, Paulo G, Vano E, Holmberg O (2022). Strengthening radiation protection education and training of health professionals: conclusions from an IAEA meeting. J Radiol Prot.

[17] European Commission (2012). Radiation Protection n^o^ 162, Criteria for Acceptability of Medical. Radiological Equipment used in Diagnostic. Radiology, Nuclear Medicine and Radiotherapy. Available from: https://ec.europa.eu/energy/sites/ener/files/documents/162.pdf

[18] European Commission (2018) Radiation Protection n^o^ 185, European Guidelines on Diagnostic Reference Levels for Paediatric Imaging

[19] Impens NREN, Salomaa S (2021). The joint roadmap for radiation protection research: outreach and future. J Radiol Prot.

